# Modulation instability control via evolutionarily optimized optical seeding

**DOI:** 10.1515/nanoph-2025-0070

**Published:** 2025-06-09

**Authors:** Lynn Sader, Yassin Boussafa, Van Thuy Hoang, Raktim Haldar, Michael Kues, Benjamin Wetzel

**Affiliations:** XLIM Research Institute, CNRS UMR 7252, University of Limoges, 87060 Limoges, France; Institute of Photonics and Cluster of Excellence PhoenixD, Leibniz University Hannover, 30167 Hannover, Germany; School of Electrical and Computational Sciences (SECs), Indian Institute of Technology Bhubaneswar (IITBBS), Bhubaneswar, Odisha 752050, India

**Keywords:** nonlinear fiber optics, modulation instability, incoherent spectral broadening, spectral correlation, noise-driven processes, machine learning

## Abstract

Controlling nonlinear pulse propagation in optical fibers is paramount for applications spanning spectroscopy and optical communication networks. However, the inherent complexity of laser pulse evolution in matter, shaped by the interplay of nonlinearity and dispersion, poses significant challenges in experimental situations. Modulation instability, a fundamental process in nonlinear fiber optics, illustrates such experimental issues due to its noise-driven nature, leading to unpredictable dynamics and thus requiring advanced control strategies. Here, we investigate noise-driven modulation instability during nonlinear fiber propagation, underlining the potential of coherent optical seeding and machine learning to jointly control incoherent spectral broadening dynamics. By introducing weak coherent seeds into an initial laser pulse, we demonstrate the ability to tailor noise-driven MI properties through fine adjustments of the seed parameters driven by evolutionary algorithms. In particular, real-time spectral characterization is achieved via time-stretch dispersive Fourier transform, enabling optimized control of spectral intensity correlations. Our experimental results highlight the effectiveness of combining coherent optical seeding with optimization techniques such as genetic algorithms, to tailor incoherent spectral fluctuations arising from the competition between coherent and incoherent nonlinear frequency conversion processes. Specifically, we show that the proposed approach can be leveraged on-demand, to shape specific correlation features in the output spectrum. The implications of our research extend beyond the sheer process of modulation instability, offering promising applications in advanced optical information processing. By demonstrating simple yet robust and flexible management strategies, this work paves the way for next-generation nonlinear photonic technologies, exploiting incoherent processes in practical optical fiber architectures.

## 1 Introduction

Readily controlling nonlinear pulse propagation in optical fibers has attracted much attention over the years, for applications ranging from laser source development to biological imaging techniques [1], [2], [3]. Such applicative prospects are however associated with substantial challenges in realistic experimental conditions. In nonlinear photonics, those primarily arise from the complexity of laser pulse evolution, mediated by the conjoint effects of nonlinearity and dispersion.

In this framework, modulation instability (MI) constitutes a ubiquitous and fundamental phenomenon of nonlinear fiber optics, as well as a prime example of complex dynamics arising from the interplay between Kerr nonlinearity and (typically anomalous) dispersion. Although MI can be harnessed advantageously in nonlinear applications, it can also be a major source of signal fluctuations and distortion. For instance, under specific conditions, the evolution of long pulses (i.e., from picosecond to continuous wave laser) can be significantly impacted by noise effects amplified by modulation instability [4], [5], [6]. In many optical fiber architectures, spontaneous (i.e. noise-driven) MI that arises from quantum or technical noise (e.g. laser intensity fluctuations) can thus severely degrade the overall performance of the system or lead to substantial instabilities in the output signal (e.g. incoherent spectral broadening, extreme wave formation, etc.). As fiber-based lasers and systems continue to expand with increased complexity, controlling or suppressing MI has thus become an active field of research, pursued to mitigate inherent signal fluctuations and therefore expand the range of practical applications, such as in optical communications networks [7], [8], [9]. Importantly, MI is also a fundamental process occurring at the onset stage of nonlinear fiber propagation, typically leveraged to trigger significant spectral broadening and cascaded frequency conversion processes for e.g. parametric amplification, supercontinuum [6] and frequency comb generation [10]. In the spectral domain, MI yields the formation of symmetric spectral sidebands on both sides of the input pump signal, resulting from the exponential growth of weak perturbations during propagation [5]. Contrariwise, in the temporal domain, MI leads to the formation of localized (breather-like) structures [11], [12] widely-known to influence subsequent nonlinear dynamics during fiber pulse propagation (i.e. pulse break up, soliton fission, dispersive wave formation and Raman self-frequency shift) [13]. Altogether, its detrimental impact on linear systems and its strong potential in tailoring nonlinear pulse evolution underscores the critical importance of controlling MI dynamics for the advancement of modern photonic systems.

From an analytical viewpoint, the nonlinear dynamics and evolution of MI components have been thoroughly studied via a variety of models (three wave truncation, undepleted pump, Akhmediev breather theory) with different approximations depending on the studied MI regime [4], [11], [14], [15]. However, when dealing with noise-driven (spontaneous) MI, the impact of noise and the high-sensitivity of the system to the initial conditions generally imposes the use of numerical simulations or advanced experimental techniques to gain insight into such inherently incoherent dynamics [12], [16], [17], [18], [19], [20]. For almost two decades, characterizing MI dynamics and understanding related noise-driven phenomena has been instrumental to many fields of photonics spanning the generation of ultrafast pulse trains or localized temporal structures [6], [21], [22], the tailored collision of soliton-like pulses [23], [24], the study of Fermi-Pasta-Ulam reversibility [14], [25] or the statistical study of extreme event formation (i.e. optical rogue waves) [12], [26].

In this context, several approaches have been developed to suitably adjust and inform the design of MI propagation regimes. Dispersion management and specialty fibers (with e.g. longitudinal variation of propagation properties) have been previously investigated and successfully developed [27]. Conversely, the adjustment of the initial conditions involving the laser source tunability (e.g., peak power, pulse shape) has been investigated for a variety of MI propagation scenarios [28], [29], [30], [31]. However, these methods are not always suitable for effectively controlling MI, as it is challenging to dynamically adjust pulse propagation conditions towards the desired output characteristics. In particular, adjusting spectral fluctuations properties and specifically tailoring the statistical correlations between different spectral components are hardly possible with standard methods [16], [18], [32], [33], [34], [35], especially when the propagation dynamics are extremely sensitive to noise, such as at the fringe between coherent and noise-driven MI regimes [19].

Optical seeding, wherein an optical perturbation is introduced to influence the propagation dynamics has thus emerged as a powerful technique to gain versatile control on both coherent and incoherent MI processes [36]. Previous work has explored MI seeding, for example in idealized scenarios like four-wave mixing (FWM) dynamics [37], [38] or to excite breather collisions [23], [24], where the process is more predictable and manageable from either an analytical or numerical viewpoint. Optical seeding was also explored in a noise-driven regime, leveraging the high sensitivity of spontaneous MI dynamics to initial conditions: weak initial seeds, with adjustable wavelengths, intensities, and phases, can introduce an initial modulation of the pump signal, ultimately leading to modified MI characteristics. Relying upon this concept, properties of MI-driven supercontinuum generation triggered by weak initial seeds have been studied both numerically and experimentally. These investigations have employed continuous-wave (CW) or quasi-CW signals [39], [40], dual pulses [41], modulated signals [42], and even incoherent seeds filtered out from an external broadband amplified spontaneous emission (ASE) source [43]. However, under experimental conditions, where spontaneous MI and noise are present, achieving fine-tuned control over the dynamics becomes considerably more complex. In this case, the challenge lies in efficiently identifying the optimal seed parameters within the highly complex landscape of nonlinear propagation dynamics, to ultimately achieve incoherently broadened signals with the desired characteristics.

Within this framework, the emergence of machine learning (ML) techniques holds transformative potential for understanding and controlling MI processes. ML has been extensively adopted across various scientific and technological fields to harness complex systems, thanks to its exceptional ability to analyze large datasets and enhance processes such as classification, prediction, and optimization. In ultrafast nonlinear photonics, ML has been widely and effectively applied to tasks such as adapting the shape of laser pulses [44], analyzing instabilities in noise-like pulse lasers [45], optimizing the spectro-temporal properties of supercontinuum generation [3], [46], [47], [48], [49], and controlling spatiotemporal nonlinearities in multimode fibers [50]. Furthermore, ML has been successfully used to accurately compute localized MI peaks in the time domain, derived from experimental frequency-domain data, thus enabling precise characterization of unstable spectral and temporal profiles of picosecond MI [51].

By analyzing complex, high-dimensional datasets, ML algorithms are expected to predict and optimize complex dynamics such as noise-driven MI. When combined with advanced experimental characterization techniques, ML has the potential to unlock unprecedented capabilities in nonlinear optics, paving the way for self-optimizing systems and facilitating real-time control strategies for target applications [1], [46], [52], [53].

In this work, we experimentally investigate and optimize the properties of MI using the concept of weak optical seeding combined with machine learning strategies. MI is controlled by introducing two coherent seeds derived from a single initial femtosecond input pulse, whose phases and wavelengths can be independently and simply adjusted via a programable spectral pulse-shaper. This flexible yet manageable manipulation of MI dynamics can effectively impact noise-driven MI phenomena, as previously demonstrated in both guided-wave and free-space optical systems. In our fiber-based setup, however, coherent seeding is obtained by spectrally filtering a broadband laser source, providing a simpler and more scalable method compared to traditional incoherent techniques that typically involve filtered amplified spontaneous emission (ASE) noise or multiple independent lasers combined together. In fact, programmable spectral pulse-shaping allows precise, independent tuning of each seed’s properties, significantly enhancing scalability over conventional electro-optic modulation techniques, which typically require separate modulators or complex multi-seed setups.

In our setup, the broadened output spectra are monitored using an optical spectrum analyzer for averaged spectral measurements. However, to monitor incoherent spectral fluctuations and characterize the spectral correlation of MI sidebands, we employ a time-stretch dispersive Fourier transform (DFT) system [18], [19], [20]. In this regime, given the intrinsic competition between weakly-seeded and spontaneous MI processes (driving different nonlinear dynamics), the adjustable parameter space is manageable through limited degrees of freedom (i.e. seed parameters) but difficult to optimize via conventional methods which tend to be overly sensitive to initial conditions and experimental perturbations. To mitigate this issue, we leveraged metaheuristic search methods, and, in particular, a genetic algorithm (GA). Paired with DFT characterization, the GA can efficiently explore the complex parameter space, enabling robust and iterative optimization of spectral correlations that deterministic or gradient-based methods may often struggle with. This approach thus allows to reliably identifying seed parameters that maximize targeted spectral features.

In this study, we thus explore the frontier between coherent and incoherent MI, where the interplay between these regimes governs the spectral broadening and evolution of the system. In particular, we propose and further detail below our experimental approach combining tailored coherent optical seeding and machine learning to iteratively tune and refine incoherent MI broadening process.

## 2 Experimental setup

**Figure 1:**
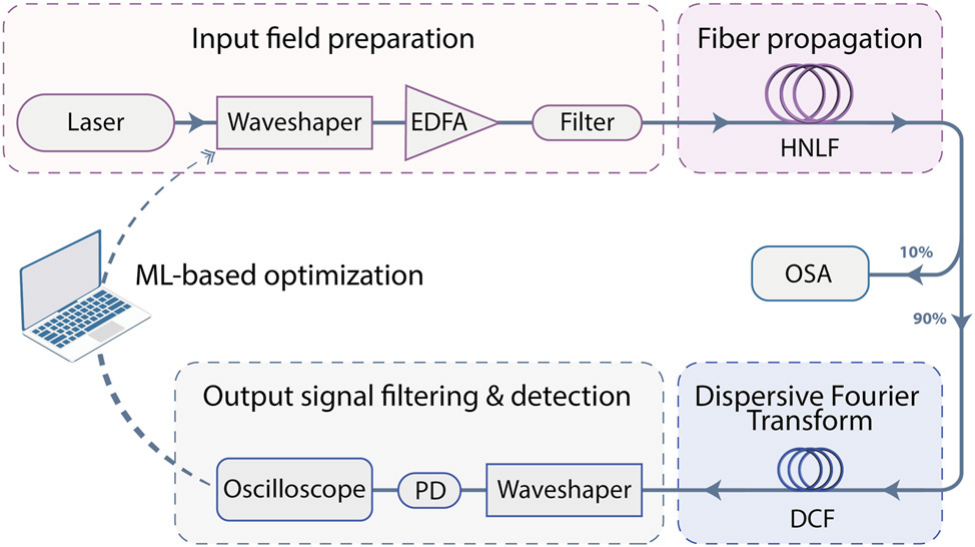
Experimental setup for optimizing incoherent modulation instability dynamics via coherent optical seeding along with machine learning iterative control. (EDFA: erbium-doped fiber amplifier; HNLF: highly nonlinear fiber; OSA: optical spectrum analyzer; DCF: dispersion compensating fiber; PD: photodiode).

The experimental setup, illustrated in Figure 1, is designed to investigate modulation instability control using coherent optical seeding and machine learning optimization techniques.

**Figure 2:**
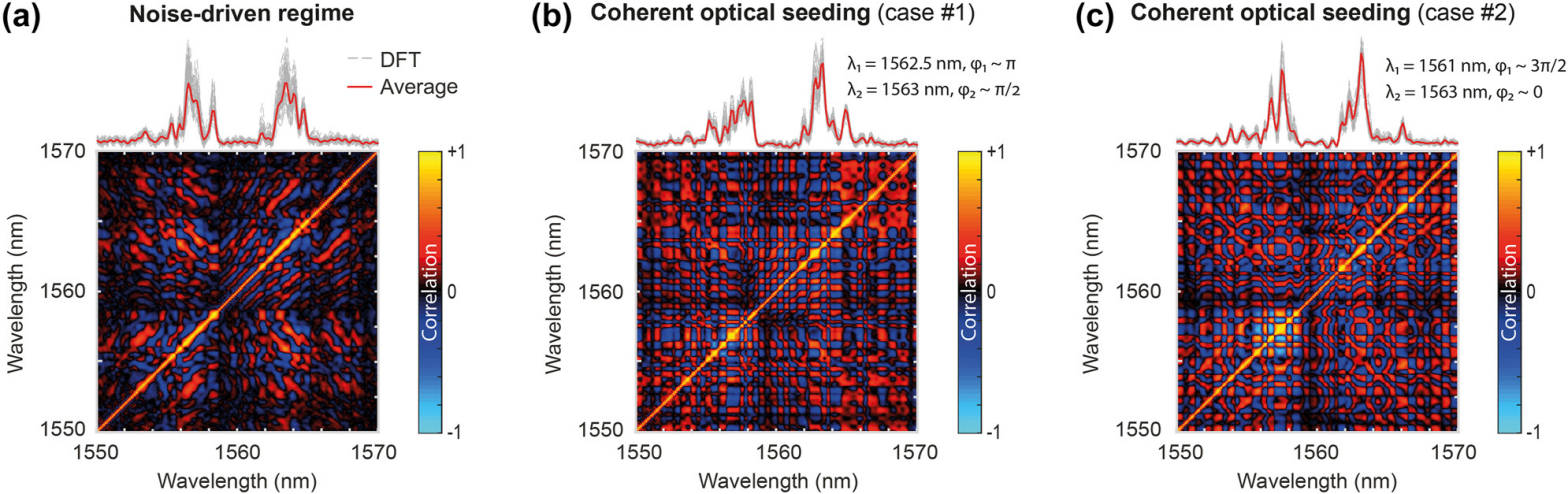
Experimental results illustrating the qualitative difference of incoherent spectral fluctuations measured for spectral broadening generated in (a) a noise-driven spontaneous MI regime and (b) two coherently-seeded MI regimes (shown for two different sets of seed properties, respectively). (Top) Output spectra measured via DFT acquisition after MI spectral broadening. The variability and fluctuations of 10 DFT spectra (grey lines) are illustrated and compared to the average spectrum (red line) computed from 500 DFT spectral acquisitions. (Bottom) Corresponding Pearson’s spectral intensity correlation maps, retrieved from the analysis of DFT spectra.

A femtosecond laser pulse (80 fs duration, 60 mW input power) centered at 1,560 nm is first filtered through a programmable spectral pulse-shaper (Finisar – Waveshaper 4000A): a 12 GHz Gaussian filter (full-width half-maximum – FWHM) is programmed to tailor the broadband spectrum and thus reshape the input signal into a picosecond pulse. Two coherent seed pulses are further added to this primary pump pulse, each with tunable central wavelengths and spectral phases, respectively noted as $\left(\lambda_{1},\phi_{1}\right)$ and $\left(\lambda_{2},\phi_{2}\right)$ . Although the seeds share the same duration as the main pump pulse (34 ps FWHM – Gaussians shape), their power is reduced by 35 dB (compared to the pump) to minimize their influence on the overall dynamics while still impacting the spectral broadening process (see Figure 2). Once prepared, the combined signals (pump and seeds) are amplified through an Erbium-Doped Fiber Amplifier (EDFA). To mitigate the effects of amplified spontaneous emission (ASE) generated during amplification, a bandpass filter is used to restrict the ASE to a 5 nm window, spanning 1,559.42 nm–1,564.63 nm. The nonlinear propagation subsequently occurs within a 485 m-long highly nonlinear fiber (HNLF) with dispersion parameters *β*_2_ = −1.78 ps^2^ km^−1^, *β*_3_ = 0.07 ps^3^ km^−1^ and a nonlinear parameter *γ* = 8.4 W^−1^ km^−1^. During fiber propagation, nonlinear spectral broadening occurs as a result of noise-driven MI, spontaneously generated from noise amplification and ultimately leading to cascaded FWM processes. This spectral broadening is then captured using an optical spectrum analyzer (OSA) for averaged spectra measurements. At the same time, using a 90:10 beam splitter, spectral fluctuations are experimentally recorded using DFT to perform real-time spectral measurements. Such a dispersive Fourier transform is achieved via linear propagation of the broadband waveform in a dispersion compensating fiber (DCF), used to stretch the pulse spectrum in the temporal domain with a dispersion factor *D* = 407 ps/nm. To further enhance the DFT measurement quality, a second Waveshaper (Finisar – Waveshaper 4000A/X) is used to filter out the pump after DCF propagation, improving the signal-to-noise ratio before detection by a fast 20 GHz photodiode (Thorlabs – DXM20AF) and a 6 GHz real-time oscilloscope (Rhode & Schwarz – RTO2064).

Here, our goal is to optimize the parameters of two coherent input seeds in order to either maximize or minimize the correlation coefficient $\left(\rho \right)$ between two frequency targets (*ν*_*a*_ and *ν*_*b*_) in the output spectrum. The output frequency targets (*ν*_*a*_ and *ν*_*b*_) are selected within the short wavelength side of the pump (i.e. <1,560 nm) while the input seeds are introduced on the opposite side of the pump, with wavelengths ranging from 1,560.5 nm to 1,564.6 nm, and their spectral phase adjusted from 0 to 2*π*. Here, we maintain the power of the seed constant and relatively weak, with a level comparable to the ASE noise level. However, using a genetic algorithm (GA), we iteratively adjust the seeds’ wavelengths and phases (i.e. $\left(\lambda_{1},\phi_{1}\right)$ and $\left(\lambda_{2},\phi_{2}\right)$ , respectively).

The GA runs over 30 generations, each generation populated with 50 different individuals (iterations), where the algorithm tunes the seeds’ parameters using the programmable Waveshaper (i.e. the 4 genes of each individual). After each iteration, 500 DFT spectra are collected, and the algorithm evaluates the Pearson correlation function of the shot-to-shot spectral intensity fluctuations between different spectral components (see Figure 2). This GA optimization process continues until the best seed parameters are found for the desired correlation between the two wavelengths *λ*_*a*_ and *λ*_*b*_. We note that measuring a correlation map for a single seeding case takes approximately 3.5 s (for both updating the waveshaper seed parameters and for the acquisition of 500 DFT traces from the oscilloscope), so that such a GA optimization over 30 generations (i.e. 1,500 seeding scenarios) typically take slightly below 1 h 30 min.

## 3 Impact of coherent seeding on MI dynamics

Spontaneous and induced MI regimes have been respectively studied for diverse applications and across various dynamical scenarios. In this work, we focus on the frontier between these two regimes, aiming to investigate the competition between weak (coherent) optical seeds – added to the initial pump pulse – and noise-driven (incoherent) dynamics, associated with broadband noise amplification [19]. This interplay between coherent and incoherent contributions thus influences the MI spectral broadening process and allows us to gain insight into how the seed parameters affect nonlinear propagation. We recall that MI spectral broadening can occur spontaneously, driven by broadband noise amplification and without any initial modulation imprinted into the input waveform. In such a case, both the properties of the pump laser (typically a long > ps pulse or a quasi-CW signal) and the fiber (or waveguide) play significant roles in MI propagation dynamics, where dispersion and nonlinear effects interact to generate new frequencies and trigger spectral broadening [27]. The introduction of coherent seeds into this spontaneous (noise-driven) MI process thus provides a straightforward method for controlling incoherent broadening dynamics.

By precisely tuning the seed properties, such as frequency, phase, and potentially amplitude, one can create a unique toolbox to adjust desired spectral features and correlations, offering advantages over classical modulation techniques that may lack coherence and scalability [29], [42]. In fact, the interaction of multiple seeds introduces additional complexity, leading to nonlinear dynamics sensitive to a broader range of parameters [54], [55]. However, this complexity also enhances our control over the MI broadening dynamics, allowing for selected stabilization and/or the achievement of desired correlation properties between various spectral components [18], [19], [33]. Notably, even dual-seed interactions can yield intricate spectral patterns, characterized by structured MI sidebands and unique correlation features.

In Figure 2, we present experimental results to underline the impact of coherent optical seeding with respect to a purely noise-driven MI broadening scenario. Figure 2(a) illustrates the spontaneous case, where the 34 ps pump pulse travels within the HNLF in the absence of any seed. In the top panel, we show the average output MI spectrum (in red) and an illustration of the spectral fluctuations provided by 10 DFT spectra (in grey), both obtained via DFT measurements. One can observe significant shot-to-shot spectral fluctuations, with the spectral components showing irregular behaviours and low stability due to the stochastic nature of the noise-driven MI process.

In the bottom panel, we display the corresponding spectral correlation map, retrieved from DFT measurements using the Pearson’s correlation coefficient [18]:

$$\rho \left(\lambda_{a},\lambda_{b}\right)=\frac{\left\langle I\left(\lambda_{a}\right)I\left(\lambda_{b}\right)\right\rangle -\left\langle I\left(\lambda_{a}\right)\right\rangle \left\langle I\left(\lambda_{b}\right)\right\rangle }{\sqrt{\left\langle I^{2}\left(\lambda_{a}\right)\right\rangle -{\left\langle I\left(\lambda_{a}\right)\right\rangle }^{2}}\;\sqrt{\left\langle I^{2}\left(\lambda_{b}\right)\right\rangle -{\left\langle I\left(\lambda_{b}\right)\right\rangle }^{2}}}$$

Here, the map indicates either positive (in red) or negative (in blue) spectral correlation, highlighting the statistical relationship between the intensity of each spectral component [16], [19], [32], [34], [35].

In contrast to the spontaneous regime, Figure 2(b) highlights the impact of introducing two coherent seeds into the system. The weak input seeds, placed at different wavelengths and phases relative to the pump, demonstrate how (weak) coherent modulation interacts with the inherent noise of the system, both nonlinearly amplified through MI and leading together to more complex cascaded four-wave mixing processes.

Indeed, the nonlinear interaction between the pump and the seeds – although weak – leads to the generation of additional sidebands and alters the overall spectral broadening and evolution dynamics. The seeds thus act as controlled perturbations, steering the spectral broadening in a more predictable manner. However, despite coherent seeding, the system remains highly sensitive to noise, operating at the boundary between coherent and noise-driven MI. Significant spectral fluctuation can be observed in Figure 2(b), where the output spectrum varies from one seeding scenario to another. Similarly, the correlation maps highlight different relationships and correlation patterns between spectral components, reflecting the complexity of MI in this mixed regime. In the next section, we investigate whether evolutionary algorithms can help the control and optimization of such complex MI dynamics.

## 4 Results

### 4.1 MI optimization via genetic algorithms

In a first experiment, we aim to leverage a genetic algorithm to optimize the correlation features between two different frequency components, i.e. a single pixel in the output spectral correlation maps shown in Figure 2(b). Specifically using the setup of Figure 1, GA optimization was performed for five different pairs of frequency targets (*ν*_*a*_ and *ν*_*b*_) located within the MI gain region (on the shorter wavelength side of the pump). The frequency detuning between the target frequencies and the pump (Δ*ν*_*a*_ = *ν*_pump_ − *ν*_*a*_ and Δ*ν*_*b*_ = *ν*_pump_ − *ν*_*b*_, respectively) was defined by a commensurability relationship *α* so that:

$$\alpha =\frac{\Delta \nu_{b}}{\Delta \nu_{a}}$$

For this study, we selected representative cases where *α* = 1.5, 2, 3 and −1 (i.e. Δ*ν*_*b*_ = 1.5Δ*ν*_*a*_, Δ*ν*_*b*_ = 2Δ*ν*_*a*_, Δ*ν*_*b*_ = 3Δ*ν*_*a*_, and Δ*ν*_*b*_ = −Δ*ν*_*a*_). Here, we first focus on two specific scenarios towards a detailed analysis: (i) the case *α* = 2, when Δ*ν*_*a*_ = 225 GHz and Δ*ν*_*b*_ = 450 GHz, and (ii) the case *α* = 1.5, when Δ*ν*_*a*_ = 300 GHz and Δ*ν*_*b*_ = 450 GHz.

**Figure 3:**
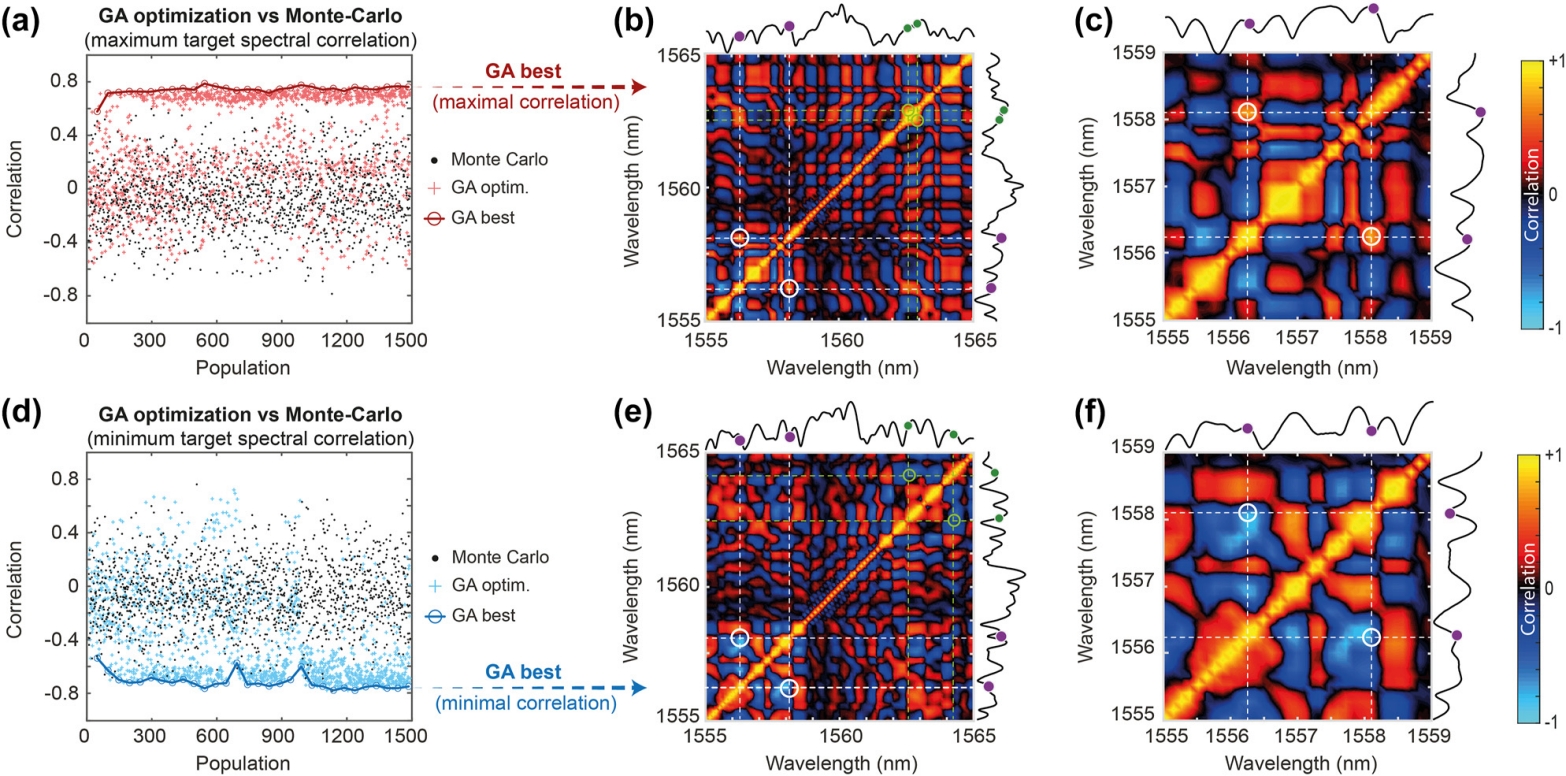
GA optimization of the correlation between two frequency targets with parameter *α* = 2 (with Δ*ν*_*a*_ = 225 GHz and Δ*ν*_*b*_ = 450 GHz). The GA evolution of the best values obtained in each generation are presented when (a) maximizing the correlation and (d) minimizing the correlation. In each case, the GA distribution results (stars) and best cases (open circle) are compared with Monte-Carlo measurements (dots) obtained by generating random input seed parameters. GA-optimized spectrum and correlation maps with the seed parameters (*λ*_1_, *ϕ*_1_) and (*λ*_2_, *ϕ*_2_) obtained for a (b) maximal correlation and (e) minimal correlation $\rho \left(\lambda_{a},\lambda_{b}\right)$ . The target optimization wavelengths are shown with white circles on the correlation maps (purple dots on the spectra) while the seed locations are highlighted with green circles on the map (green dots in the spectra). A zoom on the corresponding correlation maps around the target region of interest is shown in panels (c) and (f), respectively.

In Figure 3, we present the GA optimization results obtained for the first case *α* = 2 $\left(\Delta \nu_{b}=2\Delta \nu_{a}\right)$ , where both maximization and minimization of the correlation were performed.

In Figure 3(a), we show the evolution of the GA for 30 generations (with a population of 50 individuals each). The GA effectively optimized the correlation between the selected spectral components, achieving a maximum correlation of $\rho \left(\lambda_{a},\lambda_{b}\right)$ = 0.78 (red circles). Interestingly, the GA rapidly converged, identifying optimal seed parameters early in the process, after only a few generations. Conversely, for the minimization of the correlation displayed in Figure 3(d), the algorithm also yields significant optimization and reached a minimum value of $\rho \left(\lambda_{a},\lambda_{b}\right)$ = −0.77 (blue circles), despite some instability in the GA evolution.

To further validate the GA optimization and establish a baseline in our experiments, we conducted a Monte-Carlo (MC) analysis by performing DFT correlation measurements while the input seed wavelengths and phases were randomly varied (black dots) within the same parameter space as the GA (i.e. seed parameter boundaries). As seen in Figure 3, the GA approach (red and blue) outperformed the MC method (black dots), offering more efficient exploration of the parameter space and greater robustness against experimental noise.

In Figure 3(b) and (e), we show the Pearson’s spectral correlation maps respectively obtained after GA optimization. The white circles in the map highlight the location of the target spectral correlation regions, which offer a clear visualization of the pattern adjustment and the optimization obtained by the GA. For both optimizations, a zoom on the correlation map around this spectral region is provided in Figure 3(c) and (f), respectively.

For clarity, Figure 3(b) and (c) highlights the optimal seed parameters found by the GA (in green) $\left(\lambda_{1},\phi_{1}\right)$ ∼ (1,562.6 nm, *π*) and $\left(\lambda_{2},\phi_{2}\right)$ ∼ (1,562.9 nm, 0.6*π*) that resulted in a strong positive correlation (in red) between the target wavelengths (white circles). In contrast, the minimization case shows a clear anticorrelation signature (in blue) at the same location in the map, with the optimal seed parameters given as $\left(\lambda_{1},\phi_{1}\right)$ ∼ (1,562.5 nm, 0) and $\left(\lambda_{2},\phi_{2}\right)$ ∼ (1,564.3 nm, 1.3*π*).

We note that both cases demonstrate that the GA identifies well-defined seed parameters to effectively control the modulation instability process. However, we observe considerable variability in the seed parameters depending on the optimization target, thus suggesting that complex dynamics involving both seed wavelength and phase indeed contribute to shaping incoherent nonlinear effects during fiber propagation.

This optimization thus provides valuable insights into noise-driven MI dynamics, and privileged energy exchange mechanisms between different and competing FWM processes. However, it is worth underlining the example presented in Figure 3 corresponds to a rather simple case, where the detuning of the target frequencies is commensurate $\left(\nu_{b}=2\Delta \nu_{a}\right)$ , and thus allows (in principle) for a direct cascaded FWM to optimize the desired correlation features.

**Figure 4:**
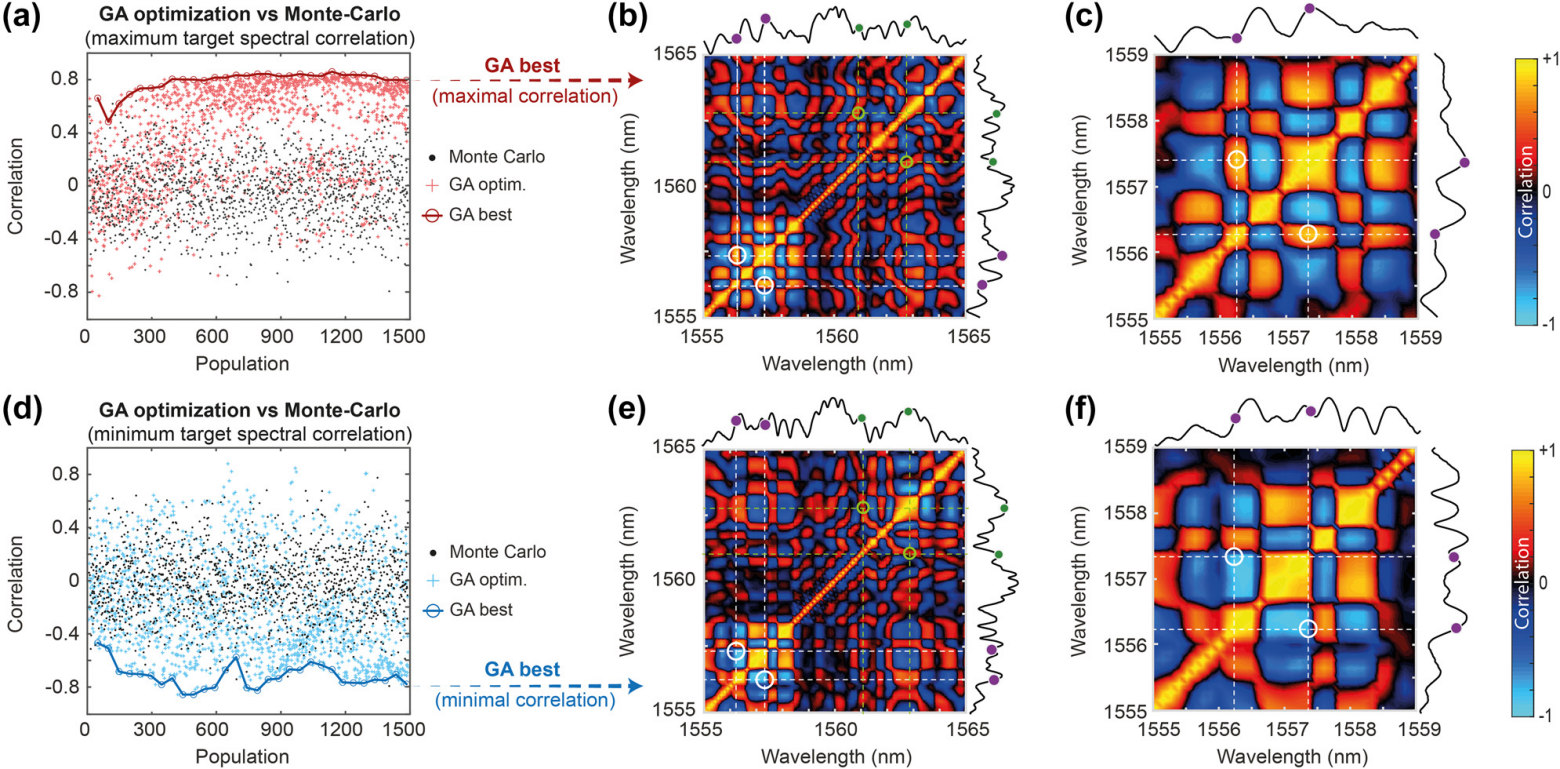
GA optimization of the correlation between two frequency targets with parameter *α* = 1.5 (with Δ*ν*_*a*_ = 300 GHz and Δ*ν*_*b*_ = 450 GHz). The content of the panels is otherwise the same as displayed in Figure 3.

In Figure 4, we thus present another optimization performed for the case where *α* = 1.5 (i.e. Δ*ν*_*b*_ = 1.5Δ*ν*_*a*_). In this regime, the optimization process is expected to prove more complex due to the lack of a direct FWM link between the target spectral components. Nevertheless, as illustrated in Figure 4, the GA achieved a maximum correlation of *ρ* ≈ 0.85 with seed parameters $\left(\lambda_{1},\phi_{1}\right)$ = (1,561.0 nm, 1.6*π*) and $\left(\lambda_{2},\phi_{2}\right)$ = (1,562.9 nm, 0), while the GA minimization reached *ρ* ≈ −0.85 by adding two coherent seeds with parameters $\left(\lambda_{1},\phi_{1}\right)$ = (1,561.1 nm, 0.33*π*) and $\left(\lambda_{2},\phi_{2}\right)$ = (1,562.9 nm, 1.66*π*). Here, we see that while the seeds present very similar wavelengths for both optimization cases, their respective phases are drastically different and responsible for a diametral change of the correlation values at the target wavelengths.

These experimental results confirm that in this case, the GA converges rather quickly and efficiently toward the desired correlation targets. We note however that maximizing correlations appears easier (and more robust) in this regime compared to correlation minimization. In the next sections, we discuss in more detail the optimization of such MI dynamics and its limitations.

### 4.2 Insight on GA-optimized seed properties

**Figure 5:**
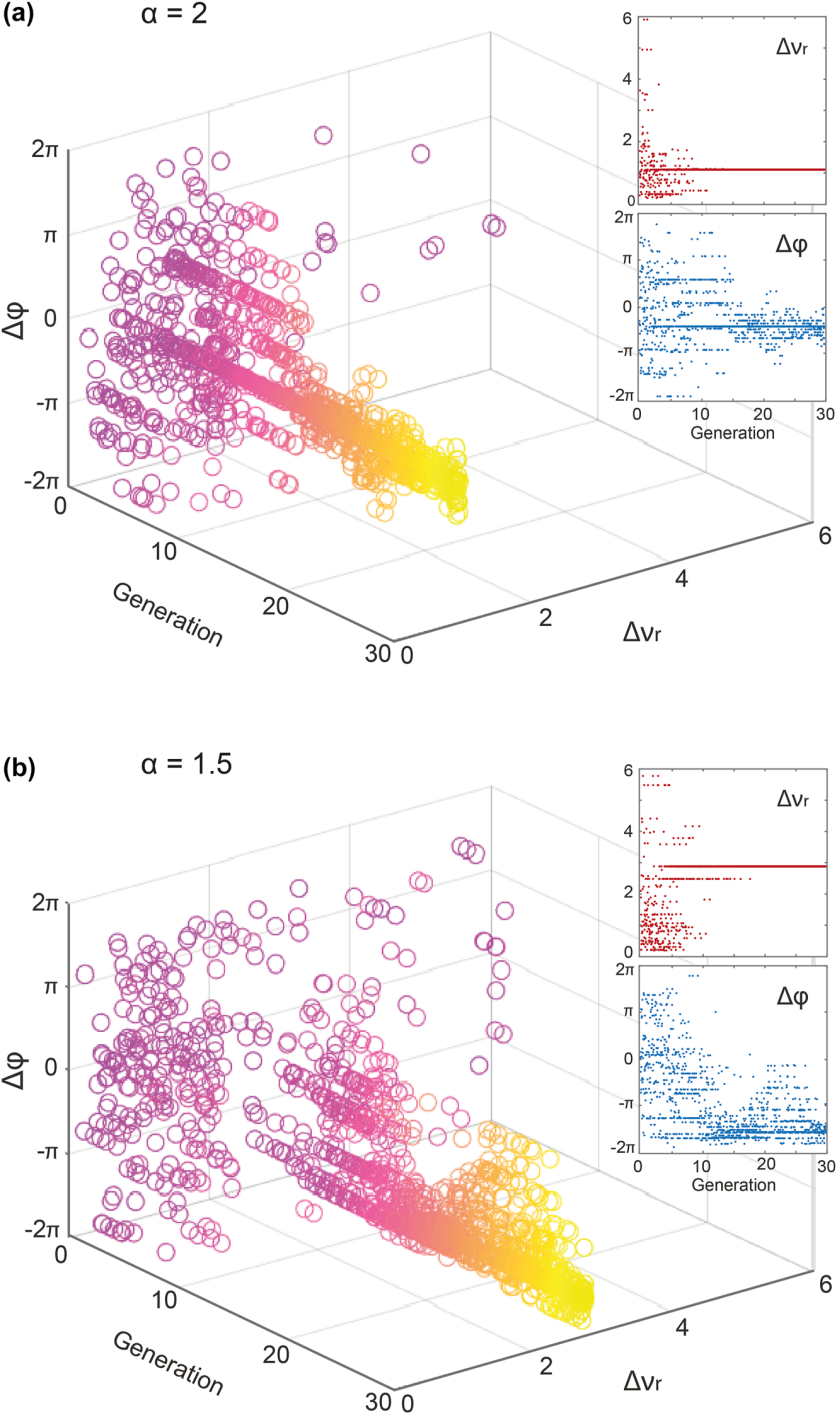
Evolution of the input seed parameters during GA optimization. The results are illustrated as a 3D representation of both the frequency detuning ratio Δ*ν*_*r*_ and the phase difference Δ*ϕ* between the two seeds. These two parameters are illustrated for all the cases tested by the GA while optimizing the maximal correlation for (a) the case *α* = 2, corresponding to Δ*ν*_*a*_ = 225 GHz and Δ*ν*_*b*_ = 450 GHz, and (b) the case *α* = 1.5 with Δ*ν*_*a*_ = 300 GHz and Δ*ν*_*b*_ = 450 GHz. The insets respectively correspond to the evolution frequency detuning ratio Δ*ν*_*r*_ and the phase difference Δ*ϕ* as a function of the GA generations.

In this study, the critical parameters influencing the dynamics of modulation instability are the frequencies and phases of the two seeds. By examining the evolution and relationship between these four parameters during the GA optimization process, one is expected to gain insight into the seed parameters and their impact on the competing processes occurring during propagation in the HNLF. As interpreting a 4D parameter space is visually difficult, we here focused on analyzing the evolution of the frequency detuning ratio between the seeds, Δ*ν*_*r*_ = Δ*ν*_2_/Δ*ν*_1_, and their corresponding phase difference, Δ*ϕ* = *ϕ*_2_ − *ϕ*_1_. In Figure 5, we illustrate the evolution of these seed parameters (following this change of variables) for two cases of GA optimization. Note that while a 2*π* range is sufficient for uniquely defining a phase difference, the 4*π* range presented in Figure 5 originates from independently varying the absolute phases of the two seeds (each over [−*π*, *π*]) before computing their difference, thereby avoiding phase unwrapping operations. This broader visualization facilitates clearer insight into both GA convergence behaviour and potential degeneracies originating from competing FWM processes (see also Table 1 for absolute phase values after GA optimization).

**Table 1:** Summary of experimental results: comparison of optimized correlation in the output spectrum for different target wavelengths. The optimized seed parameters and corresponding correlation after GA optimization (i.e. 1,500 cases with different seed parameters) are also shown for completeness.

Commensurability			Optimization target	Optimized input seed parameters				Optimized correlation
*α*	Δ*ν*_*a*_	Δ*ν*_*b*_		$\lambda_{1}\;\left[\mathrm{nm}\right]$	$\phi_{1}\;\left[\pi \right]$	$\lambda_{2}\;\left[\mathrm{nm}\right]$	$\phi_{2}\;\left[\pi \right]$	$\rho \left(\lambda_{a},\lambda_{b}\right)$
1.5	300 GHz	450 GHz	Maximize	1,561.0	1.603	1,562.9	0.016	**0.85**
			Minimize	1,561.1	0.330	1,562.9	1.660	**−0.85**
1.5	225 GHz	337.5 GHz	Maximize	1,561.0	1.985	1,564.3	0.475	**0.75**
			Minimize	1,560.7	1.935	1,562.4	0.612	**−0.85**
2	225 GHz	450 GHz	Maximize	1,562.6	1.051	1,562.9	0.603	**0.78**
			Minimize	1,562.5	0.026	1,564.3	1.326	**−0.77**
3	200 GHz	600 GHz	Maximize	1,561.8	1.272	1,563.6	1.374	**0.72**
			Minimize	1,561.5	0.862	1,562.7	0.168	**−0.81**
−1	450 GHz	−450 GHz	Maximize	1,562.5	0.800	1,563.0	1.560	**0.81**
			Minimize	1,563.2	1.375	1,564.0	0.075	**−0.84**

Bold values correspond to “optimized spectral correlation” values between selected wavelength λ_a_ and λ_b_ after GA optimization.

In Figure 5(a), we present the evolution of these variables for the case *α* = 2 over 30 generations of the GA (i.e. Δ*ν*_*a*_ = 225 GHz and Δ*ν*_*b*_ = 450 GHz). As can be seen, both the phase difference Δ*ϕ* and frequency detuning ratio Δ*ν*_*r*_ converge progressively, transitioning from an initial random exploration of the parameter space to well-defined values of Δ*ϕ* and Δ*ν*_*r*_. This can be further visualized from the insets, where we observe a fast and clear convergence of the frequency detuning ratio Δ*ν*_*r*_ (red dots) toward an optimal value at the onset of the GA (within only the first ∼10 generations). We observe that the frequency detuning ratio stabilizes around Δ*ν*_*r*_ ∼ 1, which is not unexpected, as it closely matches a sub-harmonic of the commensurability relationship between the target frequencies (i.e. *α* = Δ*ν*_*b*_/Δ*ν*_*a*_ = 2). On the other side, the evolution of the phase difference Δ*ϕ* (shown with blue dots in the inset), exhibits more variability and only begins to converge after approximately 15 generations. Although the phase difference ultimately converges to around −*π*/2, its optimization is not as straightforward as compared to the frequency detuning.

While this behavior might appear trivial, it offers insights into the MI mechanisms driven by four-wave mixing. For instance, in the case where *α* = 1.5, shown in Figure 5(b), the convergence of the GA is more complex. The 3D plot displays how the relationship between frequency detuning and phase evolves over the GA process, highlighting the challenge of achieving robust convergence within the first 20 generations. As illustrated in the insets of Figure 5(b), the frequency detuning ratio of the seeds converges more slowly, only reaching Δ*ν*_*r*_ ∼ 3, after approximately 20 generations. Additionally, the phase difference optimization, in the bottom subset, exhibits a rather chaotic behavior, requiring nearly 27 generations to reach the optimal phase difference of Δ*ϕ* ∼ −1.5*π*.

In Figure 5, the GA convergences are only presented for two correlation maximization cases, but similar trends were observed across all five combinations of target frequencies, for both correlation maximization and minimization scenarios. The frequency detuning ratio typically converges quickly, while the phase optimization takes longer and demonstrates more complex patterns. For completeness, we further provide a summary of such GA-based optimization results of spectral correlation in Table 1.

It is worth noting that the underlying reason for these frequency and phase dependencies lies in the nature of MI and four-wave mixing: MI constitutes a degenerate FWM process, where energy is transferred from the pump to unstable frequency components. When considering induced MI, which is optically-seeded, the frequency relationship between the different components is governed by the energy conservation condition:

$$2\nu_{\text{pump}}-\nu_{+}-\nu_{-}=0$$

In addition, depending on the appropriate phase-matching conditions, energy can either deplete the pump and flow towards the sidebands (*ν*_+_ and *ν*_−_) or flow back to the pump while depleting the sidebands [4], [15], [37]. Understanding this interplay between seed frequency and phase is therefore crucial in controlling these nonlinear effects, and thus plays a key role in optimizing spectral broadening mediated by modulation instability. However, straightforward analysis and fundamental interpretation based on perturbation theory, truncated three-wave approaches or more refined analytical approaches [4], [14], [54], [55] possess obvious limitations in such a context of weak coherent seeding, as illustrated in Figure 2.

One needs to bear in mind that, during such a propagation, the picosecond pump pulse experiences self-phase modulation (SPM) that can lift the degeneracy of MI-based FWM. Further considering multiple coherent seeded MI processes – and their respective FWM cascade – both competing dynamically with spontaneous MI noise amplification, we emphasize that addressing experimentally such complex scenario is a real challenge that can directly benefit from the use of ML approaches [1], [56]. While out of the scope of this study, we expect that, in the future, these propagation scenarios and data acquisition can leverage physics-informed strategies to gain further insight into such complex nonlinear frequency conversion processes [57], [58].

### 4.3 Discussion on evolutionary algorithm efficiency

Another point of interest lies on the selection of the algorithm used to control MI spectral fluctuations. In our study, we chose to optimize the correlation coefficient between two spectral targets using genetic algorithms, after carefully evaluating different machine learning approaches. One of the key comparisons was with particle swarm optimization (PSO), a commonly used technique in similar contexts [3], [44], [59].

**Figure 6:**
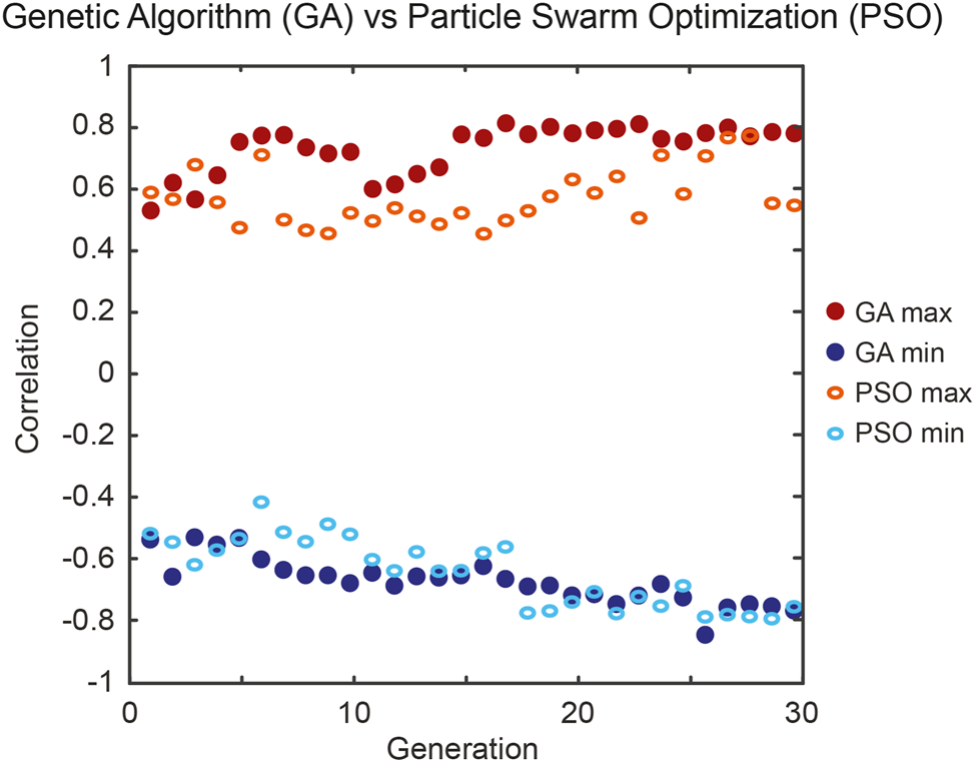
Comparison of optimization algorithms for experimentally maximizing (red) and minimizing (blue) the correlation coefficient between frequency targets with detuning Δ*ν*_*a*_ = 450 GHz and Δ*ν*_*b*_ = −450 GHz (i.e. *α* = −1). GA results are shown with plain dots and PSO results with open ovals.

Figure 6 illustrates the correlation evolution for the frequency target with detuning Δ*ν*_*b*_ = −Δ*ν*_*a*_ = −450 GHz (i.e. *α* = −1). In both maximization and minimization scenarios, GA showed a more consistent and rapid convergence toward the optimal solutions, efficiently reaching the desired values within fewer generations. While very similar in terms of convergence, PSO exhibited more irregular behavior, with greater variability in the early stages of optimization, and requiring a longer settling time towards an optimal result.

In the examples shown in Figure 6, we observe that the first generation of both GA and PSO already yield a target correlation of $\left|\rho \right|∼0.5$ , herein obtained by a mere post-selection of the initial parameter space in the search algorithm. As the optimization is performed over 30 generations, both approaches lead to a significant improvement of the target correlation function to reach $\left|\rho \right|∼0.8$ (for both correlation maximization and minimization).

Compared to a Monte Carlo approach, which relies on the random exploration of the parameter space and here typically leads to a distribution centered around *ρ* ∼ 0 (see Figures 3 and 4), both optimization algorithms worked fairly efficiently. In our experimental study, GA consistently provided a more structured and reliable optimization than PSO, avoiding local extrema and yielding slightly faster convergence. We however note that the optimization and convergence of algorithms illustrated in Figure 6 seem to be primarily bounded by the experimental stability of the DFT acquisition, as well as the underlying sensitivity of these nonlinear processes to the initial conditions (pump intensity noise, EDFA thermal stability, etc.). In this regime, while further parameter tuning could potentially improve PSO’s stability, our findings highlight the GA’s effectiveness and robustness in efficiently navigating the parameter space towards a successful incoherent signal optimization.

## 5 Conclusions

Our study highlights the critical role that coherent optical seeding can play in controlling incoherent nonlinear processes. In particular, we experimentally demonstrate the impact of the input seed parameters – both frequency and phase – to tailor noise-driven modulation instability in nonlinear fiber propagation. By optimizing these initial parameters through machine learning techniques, we were able to achieve effective control on the MI broadening dynamics during nonlinear fiber propagation and the spectral fluctuation properties at the fiber output. Unlike previous studies relying on static, incoherent, or idealized seeding schemes, our approach demonstrates online experimental control of noise-driven MI dynamics using weak coherent seeds optimized. By shaping spectral correlations “on-demand” by means of a genetic algorithm, while probing the interplay between coherent seeding and stochastic fluctuations, our method provides experimental insights into nonlinear fiber propagation that extend beyond the capabilities of standard numerical modeling, offering a practical advance toward self-optimized nonlinear photonic systems.

Specifically, by suitably adjusting input coherent signals (with a waveshaper), and implementing real-time spectral characterization of the incoherent output signal (via time-stretch dispersive Fourier transform), we showed the potential of our experimental approach to tailor and optimize intensity correlations between specific spectral components in the fluctuating output spectra. We emphasize that, in the current work, the limited range of achievable correlation values (|*ρ*| typically below 0.85) primarily stems from the intrinsic stochasticity of the MI dynamics studied, at the frontier between coherent and incoherent regimes (see Figure 2). While experimental factors like laser and amplifier noise, DFT measurement uncertainties, and overall system stability play a role, the main limitation arises from the underlying dynamics, as attested from the bounded correlation values also observed through Monte–Carlo probing of the parameter space (see Figures 3 and 4). Although using stronger seeds could suppress noise and thus push correlation features closer to unity – characteristic of induced MI dynamics – our study deliberately focuses on a regime where weak seeding competes with spontaneous noise-driven processes, ensuring a non-trivial interplay that inherently constrains the maximum achievable correlation.

While this work focused on a rather straightforward evolutionary algorithm for optimizations (i.e. GA and PSO [1], [3], [59]), future studies will explore advanced computational methods such as deep learning [56] and physics-informed techniques [58], [60] to predict optimal input signal properties within a wider parameters space while gaining insight into the underlying dynamics of weakly seeded noise-driven processes such as MI. We expect these approaches, extending beyond iterative techniques, to have the potential in enhancing both the accuracy and efficiency of optimization processes while opening new possibilities for fine-tuning nonlinear dynamics.

In fact, numerical modelling may further advance our understanding of advanced seed-target relationships within such complex FWM processes. However, the sensitivity of the dynamics to the initial conditions constitutes a significant challenge to obtain more than a qualitative agreement between numerical simulations and experiments. Yet, the empirical success of our approach underscores the potential of real-time optimization in architectures relying on complex and incoherent nonlinear dynamics. From a practical viewpoint, our implementation may relax the constraints on the non-specialist user while potentially improving the precision of experimental outcomes. Indeed, although our investigations focus on the specific case of MI, the implications of this research extend to broader applications, with potential cross-disciplinary impacts in various fields of photonics, spanning imaging, nonlinear microscopy, spectroscopy up to quantum information processing [61].
